# Design of Biodegradable PU Textile Coating

**DOI:** 10.3390/polym16162236

**Published:** 2024-08-06

**Authors:** David De Smet, Jente Verjans, Miriam Bader, Anke Mondschein, Myriam Vanneste

**Affiliations:** 1 Centexbel, Technologiepark 70, 9052 Zwijnaarde, Belgium; 2 FILK Freiberg Institute gGmbH, Meißner Ring 1-5, 09599 Freiberg, Germany

**Keywords:** polyurethane, coating, biodegradable, textile

## Abstract

Polyurethane (PU) coatings are used in diverse applications such as textile coating. Up to today, landfilling is still the most occurring way of processing PU waste. Biodegradation is an alternative route for processing PU waste and decreases the amount of microplastics in the case of landfilling. In this study, a biodegradable PU textile coating was developed. The PU was characterized via Fourier-transformed infrared (FT-IR) thermogravimetric analysis (TGA) and differential scanning calorimetry (DSC). The PU was thermoplastic and had a melting point of approximately 33 °C. The performance of the coating was studied by assessing the water barrier and mechanical properties. The PU coating completely disintegrated, and the biodegradation of PU was assessed in soil and was almost 60%. Furthermore, the plant toxicity was examined by evaluating seedling emergence and growth.

## 1. Introduction

Polyurethane (PU) is widely used due to its versatile and superior properties. Most common applications include foams, coatings, and elastomers. At its end of life (EoL), landfilling is still the most applied way to process PU waste, since toxic byproducts could be produced during PU combustion. Different recycling routes have been explored, such as solvation, solvolysis, or debond on demand [1,2,3,4,5]. However, since landfilling of PU waste is still the state of the art, there is a need for biodegradable PU that degrades in the environment and releases no toxic products or microplastics. Conventional PU is not prone to physical, biological, or chemical degradation and can therefore not be biodegraded.

Biodegradation means the breakdown of organic substances by living organisms or enzymes resulting in a reduction in their molecular weight. In favorable conditions, it can even result in the complete mineralization of degraded material. However, the complete degradation of polymers usually requires the cooperation of several different organisms. It can consist of a few stages: the breakdown of the polymer to monomers, their reduction to simpler compounds, and final degradation to carbon dioxide, water, or methane. Biodegradation is usually more environmentally friendly than chemical degradation, as it does not require high temperatures and complicated reagents [6].

Amorphous regions of PU are more susceptible to degradation than crystalline regions due to their better accessibility [7]. Enzymes are also studied to degrade PU films. A major part of biodegradation can be assigned to the hydrolysis of the polyester fraction of polyester-based PU by esterases. Esterase E3576 shows high degradation activity toward polycaprolactone (PCL)-based PU. Combining E3576 and amidase increased the hydrolysis of urethane compared with using the enzymes separately [8,9]. The chemical structure of the diisocyanate, polyol, and chain extender, as well as the ratio of these components in PU, determine its mechanical properties, processability, and biodegradation. Only a limited number of suitable diisocyanates (e.g., hexamethylene diisocyanate (HDI) or isophorone diisocyanate) are commercially available for the formulation of biodegradable PU. HDI is widely chosen as diisocyanate for the synthesis of biodegradable PU because of the excellent mechanical properties of the resulting PU. Poly(butylene)adipate degraded much faster compared with poly(ethylene succinate) with similar molecular weight [10]. Hettrich et al. synthesized novel diisocyanates based on amino acids containing ester linkages. The resulting PU had ester linkages, making it more susceptible to hydrolysis. These mechanical properties are not those of typical PU. A softening of the PU was observed. The degradation was estimated by a hydrolysis study over 3 months. A conversion between 5 and 10% was noticed [11]. The design of chain extenders with functional groups (e.g., ester or phosphate esters) has been one of the approaches of researchers to enhance the degradation of hard segments, which are known to have limited degradation rates. A degradable chain extender based on lactic acid and ethylene glycol accelerated the degradation of the hard segment, while no considerable effect was noticed on the mechanical and thermal properties [12,13]. Chain extenders based on amino acids have also been developed to enhance the enzyme-mediated degradation of polyurethanes [14,15]. The chemical structure of the polyol also influences the degradation of PU. Common polyols used in the synthesis of biodegradable PU include PCL, poly(ethyleneglycol) PEG, poly(propyleneglycol) (PPG), polyols based on hydroxy acids such as glycolic acid, lactic acid, and their copolymers. In the case of PCL, a decrease in microbial degradation with an increase in molar mass were observed. After 30 days, biodegradation varied between 5 and 25%. Increasing PCL content and decreasing PEG content decrease hydrophilicity and thus (hydrolytic) degradation [16,17,18]. The incorporation of PEG, PPG, or polyols based on hydroxy acids increased the hydrophilicity of the PU, making it more susceptible to hydrolysis. In polyester-polyol-based biodegradable PU, the main functional groups susceptible to hydrolytic or enzymatic degradation are ester, urethane, urea, and amide, of which esters show the highest susceptibility to hydrolysis. Polyether polyols are more hydrolytically stable than polyester polyols and can be used in combination with polyester polyols to tailor the degradation rate and flexibility [19]. Covestro has a PUD, Impranil DLN-SD, which is claimed to be biodegradable. Impranil^®^ DLN-SD is a fossil-based polyurethane showing promising degradability rates when dispersed in water. It was tested for biodegradability according to the CO_2_ evolution test (OECD test standard 301) and shows a degradation of above 50% in 28 days [20]. Another application area of biodegradable PU is medical implants. Artelon™, a degradable PU, has been used clinically for cruciate ligament reconstruction and as a spacer for the trapeziometacarpal joint [21]. NovoSorb™ (PolyNovo Biomaterials) is a family of biodegradable PUs for orthopedic and dermal applications [22,23].

However, most reported applications of biodegradable PU focus on medical implants and cosmetics and not on (textile) coatings. This report describes the design of a biodegradable PU for textile coatings. Therefore, biodegradable moieties were incorporated in the PU backbone, which allows biodegradation and thus reduces the amount of microplastics in the environment in the case of landfilling.

## 2. Materials and Methods

### 2.1. Materials

Bismuth neodecanoate, dimethylol propionic acid (DMPA), lysine, triethylamine (TEA), and isophorone diisocyanate (IPDI) were bought from Sigma-Aldrich (Bornem, Belgium). Methylethyl ketone (MEK) was bought from ChemLab (Zedelgem, Belgium). Respumit NF01, Edolan XCI, and Edolan XTP were sampled by Tanatex Chemicals (Ede, The Netherlands). Polycaprolactone (PCL) polyol was supplied by Daicel (Osaka, Japan). Woven polyester fabric (105 g/m^2^) was acquired from Concordia Textiles (Waregem, Belgium).

### 2.2. Synthesis of Biodegradable PU Dispersion

A total of 80 g PCL polyol and 3.54 g DMPA were dissolved in 100 mL MEK at 50 °C under nitrogen atmosphere. After increasing the temperature to 80 °C, 0.2 g of bismuth neodecanoate and 18.5 g IPDI were added. After 4 h, the reaction was cooled down, and the solution was neutralized with TEA. Subsequently, PU was dispersed in deionized water, resulting in a milky white dispersion. MEK was removed by a rotary evaporator under reduced pressure at 40 °C. Finally, a solution of 20% lysine was added to the dispersion and stirred for 30 min. The solid content of the PU dispersion was approximately 40 wt%. The particle size distribution was characterized with a Zetasizer Nano ZS device of Malvern Instruments by dynamic light scattering (DLS). Measurements were performed at 23 °C, a material refractive index (RI) of 1.45, and with water as dispersant (RI 1.33) in backscatter mode (173°). At least twelve measurements were carried out per run. Subsequent dilutions by a factor of five were performed until stable results were obtained without particle interaction and double scattering but with sufficient scattering intensity. Four different concentrations were measured, and the most diluted sample (solids content of 0.025%) was used for analysis. Z-Average and size distribution were analyzed. Polydispersity index (PDI) was calculated according to ISO 13321:1996E [24].

### 2.3. Fabric Coating

A total of 80 g of the developed bio-based PU dispersion and 0 or 1% Edolan XCI (polyisocyanate-based crosslinker) were mixed. Respumit NF01 was added to avoid foaming, and Edolan XTP was added to increase the viscosity of the formulation. Three layers of 100 µm were coated on a polyester fabric. Between applying the different layers, the coating was dried 1 min at 110 °C and cured 2 min at 155 °C. To examine the film properties (mechanical and thermal properties) and the biodegradation of the PUD, the PUD without crosslinker was coated (1 layer of 100 µm) on a release paper.

### 2.4. Characterization

The biodegradable PU was characterized via FT-IR with a Nicolet 6700 spectrometer (ThermoFisher Scientific, Waltham, MA, USA). The thermal degradation of the developed PU was evaluated via TGA using a Q500 thermogravimetric analyzer (TA Instruments, New Castle, DE, USA). Analyses were performed in air from 30 °C to 600 °C (ramp rate of 10 °C/min). Glass transition temperature (Tg) and melt temperature (Tm) of the PU were measured via DSC analysis, using TA Instruments Discovery DSC2500 (TA Instruments, New Castle, DE, USA), in the range from −50 °C to 200 °C (heating–cooling–heating with a heating and cooling rate of 10 °C/min). All samples were conditioned at 23 °C and 50% relative humidity before DSC and TGA.

Elongation at break and tensile strength were measured according to ISO 13934-1 [25] using Instron electronic fabric tension tester (with a tension loading speed of 100 mm/min). The water barrier properties were assessed according to ISO 811 [26] with Textest FX 800 apparatus. The wash fastness was evaluated by measuring the water barrier properties after 10 domestic washing cycles at 40 °C (ISO 6330 [27]). The coated fabrics were dried at ambient temperature after washing.

A disintegration test was conducted to screen the potential biodegradation of PU coatings. Therefore, the coated films were put in a frame, and the samples were buried in soil (soil burial test BS 6085 [28]) to evaluate the resistance to microbiological deterioration in soil. The temperature of the soil was set at 28 °C and the humidity of the environment was at least 95%. The coating was visually assessed after two, four, and eight weeks.

A biodegradation test in soil following ISO 17556 [29] was performed to assess the biodegradation of the PU. Natural sieved soil was used as the inoculum. The water content was adjusted to 40–60% and pH was set at 6–8. The C:N ratio was at least 40:1. The biodegradation percentages were determined as the ratio of CO_2_ produced during the biodegradation and the maximum theoretical CO_2_ production based on the carbon content of the PU sample. The CO_2_ production from the sample was determined by subtracting the amount of CO_2_ in the blank samples from the amount of CO_2_ in the PU samples. Indeed, during aerobic degradation, organic material is converted into biomass, water, and CO_2_. Finally, an ecotoxicity screening and metal screening were conducted. Metals Zn, Cu, Ni, Cd, Pb, Hg, Cr, Mo, Se, As, and Co were analyzed with ICP-MS. The total fluoride content was determined using combustion ion chromatography. Plant toxicity was evaluated by examining seedling emergence and seedling growth of garden cress. Biodegradable PU was mixed in soil at a concentration of 1%. Fifteen seeds were sowed in 200 g test soil and reference (without biodegradable PU) soil immediately after mixing the PU in the soil and after 40 days. The test was performed in triplicate. At the end of the test, the number of plants per pot was determined, and the fresh weight was measured per pot after 1 week. The germination and plant yield of the test soil was compared with the reference soil to assess the plant toxicity. Statistical difference between mean germination and plant yield in test and reference soil was determined using the *t*-test.

## 3. Results

### 3.1. Characterization of PU Dispersion and Coating

**Figure 1:**
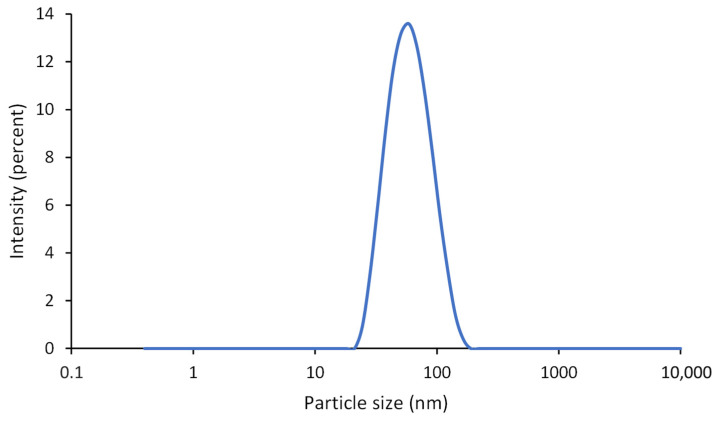
Particle size distribution by intensity of the biodegradable PU dispersion measured by DLS.

The synthesized dispersion was analyzed for particle size and particle size distribution using DLS. It showed a monomodal size distribution with a Z-Average particle size of 54 nm (Figure 1). The polydispersity index is 0.14.

**Figure 2:**
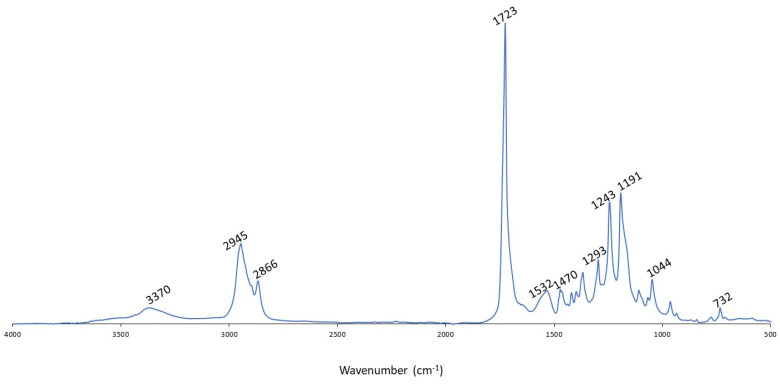
FT-IR spectrum of developed PU.

The PU coating was characterized by FT-IR. Figure 2 exhibits the FT-IR spectrum of the developed PU. The absence of isocyanate (the asymmetrical N=C=O stretch between 2250 and 2285 cm^−1^) and OH band and the presence of NH bands indicated that the PU was successfully synthesized. Characteristic bands for PU were observed in the FT-IR spectrum: NH vibration (3370 cm^−1^), NH and CN amide stretch (1532 cm^−1^), C=O urethane stretch (1723 cm^−1^), COC urethane elongation vibration (1243 cm^−1^), and COO urethane deformation vibration (732 cm^−1^). Table 1 gives an overview of the different peaks and the correlating functional groups [30,31,32,33,34].

### 3.2. Mechanical Properties of the Coating

**Figure 3:**
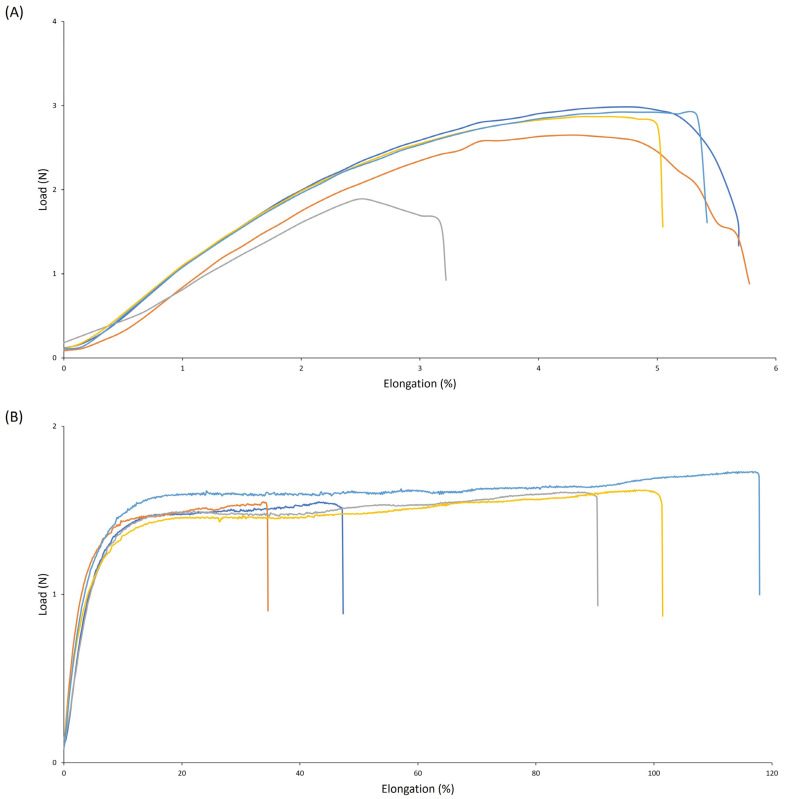
Tensile curves of biodegradable PU without (**A**) and with (**B**) crosslinker. Five replicates were measured.

The mechanical properties of the coating were assessed according to ISO 13934-1 (Figure 3). The biodegradable PU coating without crosslinker had an average elongation of 4.2% and a tensile strength of 13 MPa. Surprisingly, the addition of a crosslinker increased elongation to 76% but decreased tensile strength to 4 MPa. Generally, increasing the crosslinker increases the crosslinking density and thus creates higher rigidity, resulting in lower elongation. However, the reverse was noticed here.

The biodegradable PUD formulation with the crosslinker was coated on polyester fabric, and the water barrier properties were measured initially and after washing. The resistance to hydrostatic pressure was at least 1000 mbar. After 10 domestic washing cycles at 40 °C, the water barrier properties did not decrease, indicating that the developed coating can be applied to waterproof textiles with good wash fastness.

### 3.3. Thermal Properties of Biodegradable PU

**Figure 4:**
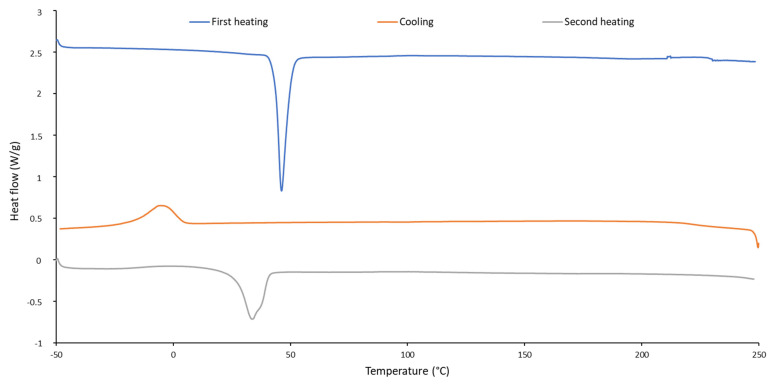
DSC analysis of biodegradable PU.

The thermal properties of the biodegradable PU were examined via DSC and TGA. During both heating runs, endotherm peaks were observed due to the melting of PU, while during cooling, an exotherm peak was noticed, which indicated the crystallization of PU. The melting point was determined during the second heating run of the DSC analysis, since the first heating run was carried out to remove volatile impurities and erase the thermal history of the polymer. Melting does not occur at one unique temperature but over a small range. The melting point was selected as the summit of the endotherm peak during the second heating run and was found to be 33 °C, which indicated that the biodegradable PU was thermoplastic (Figure 4). No glass transition was observed during the analysis. The crystallization temperature was −5 °C. The low melting point of the PU explains the tackiness of the coating.

**Figure 5:**
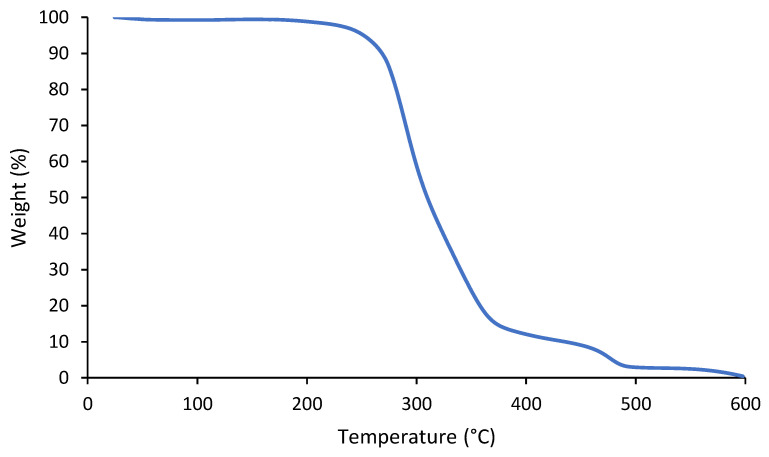
TGA of biodegradable PU.

The thermal degradation of the biodegradable PU up to 600 °C was evaluated via TGA (Figure 5). The thermal degradation started above 200 °C and almost no mass residue was measured at 600 °C. The thermal degradation occurred in three steps. First, the hard urethane segments degraded, followed by the degradation of ester groups and linear hydrocarbon chains. Finally, C=C bonds were cleaved [35,36,37].

### 3.4. Disintegration Study

**Figure 6:**
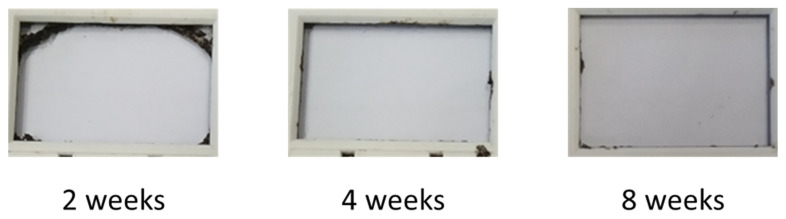
Disintegration of non-cross-linked PU film after 2, 4, and 8 weeks.

**Figure 7:**
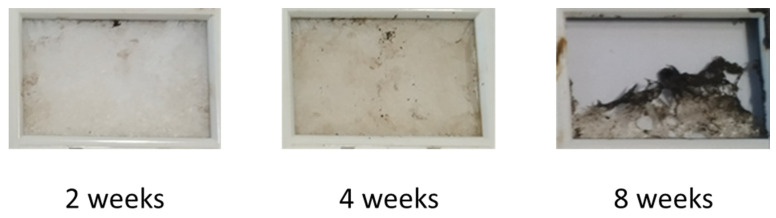
Disintegration of PU films made with 1% crosslinker, after 2, 4, and 8 weeks.

The physical disintegration of coatings was tested by burying the coatings in soil (BS 6085-2) and evaluating the disintegration after two, four, and eight weeks. Non-cross-linked coatings disintegrated fast and already showed significant disintegration after two weeks (Figure 6). The coatings were completely disintegrated after four weeks, and no small pieces bigger than 1 mm^2^ could be retrieved. Crosslinked PU coatings with a 1% crosslinker did not exhibit disintegration in the first four weeks; however, after 8 weeks, significant disintegration was observed (Figure 7). Coatings with higher levels of crosslinker did not disintegrate within 8 weeks. Indeed, by increasing the amount of crosslinker, the crosslinking density increases, and the mobility and accessibility of microorganisms in the polymer chains decreases, resulting in a decreased disintegration rate. Furthermore, crosslinking decreases the hydrolysis rate.

### 3.5. Soil Biodegradation of PU Coating

**Figure 8:**
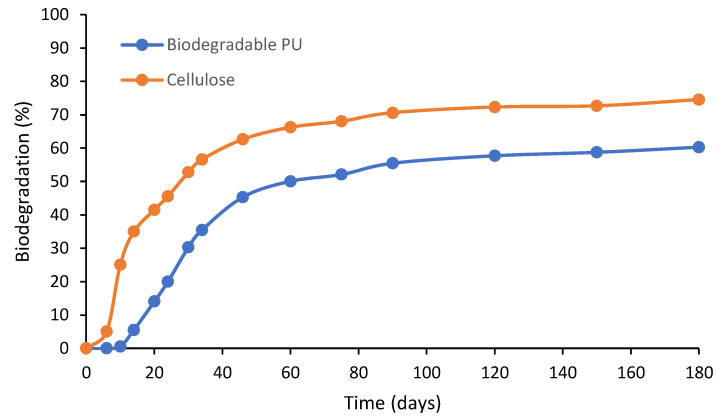
Biodegradation of cellulose and PU coating.

The biodegradation of the PU coating and reference cellulose is shown in Figure 8. The biodegradation test was stopped after 180 days. Cellulose was used as a reference material and exhibited a high biodegradation rate in the first month (up to 52%). After 6 months, cellulose was almost 75% degraded. The PU coating started to degrade after 10 days and was already degraded by approximately 50% after 2 months, after which degradation gradually increased up to 60% after 180 days. The relative biodegradation of the PU coating compared with cellulose reference amounted to 80% after 6 months.

### 3.6. Toxicity Screening

Since it is important that biodegradation does not result in environmental toxicity, the toxicity of the biodegradation residuals was screened by assessing seed germination and growth and measuring the content of certain metals and fluorine. The metal and total fluorine content are given in Table 2. Only low amounts of zinc and fluorine were measured. None of the found values exceeded the maximum limits defined by EN 13432 (European standard for the industrial compostability of packaging). The seed germination and growth of lettuce and cress seeds were assessed in soil with 1% PU and compared with reference soil without PU (Table 3). The seedlings were sowed on day 1 and after 40 days. Indeed, after 40 days, the biodegradation of PU amounted to approximately 40% and was still increasing. Thus, the potential toxic effect of biodegradation would be more pronounced in the case of seedlings sowed after 40 days. Although the germination rate and fresh weight yield were higher in soil containing PU, no significant differences in germination or growth were observed for cress seeds sowed on day 1. The germination rate of cress seeds was not significantly different in the case of seeding after 40 days for both control soil and soil with PU. Also, the fresh weight yield of cress seeds in soil containing PU did not change significantly when the seeds were seeded on the first day or after 40 days. Surprisingly, the fresh weight yield of cress in control soil increased significantly in the case of seeding after 40 days compared with day 1, which also resulted in a significant difference between fresh weight yield in control soil and soil containing PU. Indeed, it was expected that fresh weight yield would be lower due to lower fertilizer content in the soil because of leaching fertilizer over time during the wetting of the soil.

## 4. Discussion

Although conventional PU is not susceptible to biodegradation, the developed PU showed significant biodegradation, even in soil at 25 °C, which is even better compared with polylactic acid (PLA), since PLA is only industrially biodegradable. However, the biodegradation rate of crosslinked PU coatings needs to be examined. Indeed, textile coatings are often crosslinked to increase durability (e.g., wash fastness). Compared with conventional commercially available PU for textile coatings, the biodegradable PU shows minor elongation (<300%) but is still very flexible. The PU coating is hard and not tacky, which makes it suitable as a waterproof topcoat. The thermal degradation occurred in a similar way as other PU textile coatings, starting between 200–250 °C and in three steps. As expected, no mass residue was obtained at 600 °C [38]. Although the potential toxic effect of the biodegraded products was assessed by metal and fluor analysis and cress seeding and no major toxic effect was observed, future research with regard to the toxicity of the biodegraded products needs to be conducted. The PU coating contained a small amount of Zn, which was present in the polyol but complied with EN 13432.

## 5. Conclusions

A biodegradable PU was made based on PCL polyol. At their end of life (coated), textiles are often landfilled, but by using a biodegradable PU coating (preferably with a biodegradable fabric), the release of microplastics and potentially toxic products in the environment is decreased. Applications of biodegradable PU coatings include rain clothing and tents. Indeed, during usage, microplastics could be unintentionally released into the environment due to abrasion or washing. By using biodegradable coatings, microplastics from the coating will biodegrade and not accumulate, resulting in fewer microplastics in the environment. Furthermore, biodegradable (waterproof) PU coatings could also be used for products that need to degrade over time in the environment. Possible applications of intended biodegradable coatings include waterproof coatings for food packaging and body bags, antifouling coatings, and agricultural coatings (e.g., controlled-release coating for fertilizers). The non-cross-linked coating contained no heavy metals or fluorine exceeding the maximum values set by EN 13432 and showed a biodegradation rate of almost 60% after 6 months. The coating exhibited good water barrier properties and wash fastness, which makes it suitable for waterproof and wash-resistant coated textiles. DSC analysis revealed the PU had a low melting point, resulting in some tackiness at room temperature, and therefore, it can be used as an adhesive layer as well. Due to the hydrophilicity and presence of ester groups and thus hydrolysis sensitivity, the biodegradable PU coating is less suited for high technical textile applications that need to withstand hydrolysis and high wash temperatures.

## Figures and Tables

**Table 1:** Overview of functional groups in developed PU detected by FT-IR.

Wavenumber (cm^−1^)	Corresponding Group
732	COO urethane (deformation vibration)
1044	C-O stretching
1191	C-O-C ester (elongation vibration)
1243	C-O-C urethane (elongation vibration)
1293	C-C stretching
1470	CH (deformation vibration)
1532	N-H and C-N amide
1723	C=O urethane and ester (elongation vibration)
2866	CH (elongation vibration)
2945	CH (elongation vibration)
3370	NH (elongation vibration)

**Table 2:** Metal and fluorine levels in biodegradable PU coating and maximum levels as defined by EN 13432 (n.d. = not detected).

	Level in PU (ppm)	EN13432 Limit (ppm)
Zn	54.7	150
Cu	n.d.	50
Ni	n.d.	25
Cd	n.d.	0.5
Pb	n.d.	50
Hg	n.d.	0.5
Cr	n.d.	50
Mo	n.d.	1
Se	n.d.	0.75
As	n.d.	5
F	21	100

**Table 3:** Germination rate and fresh weight yield of cress seeds seeded on day 1 and on day 40.

	1st Day Seeding		40th Day Seeding	
	Germination Rate (%)	Fresh Weight (g)	Germination Rate (%)	Fresh Weight (g)
Soil + PU	93.3 ± 6.7	0.73 ± 0.02	86.7 ± 6.7	0.69 ± 0.03
Control Soil	91.1 ± 3.8	0.66 ± 0.02	88.9 ± 3.8	0.86 ± 0.09

## Data Availability

The data presented in this study are available on request from the corresponding author.
